# Egyptian Mongoose (*Herpestes ichneumon)* Gut Microbiota: Taxonomical and Functional Differences across Sex and Age Classes

**DOI:** 10.3390/microorganisms8030392

**Published:** 2020-03-11

**Authors:** André C. Pereira, Victor Bandeira, Carlos Fonseca, Mónica V. Cunha

**Affiliations:** 1 INIAV, IP- National Institute for Agrarian and Veterinary Research, 2780-157 Oeiras, Portugal; andre.c.pereira94@gmail.com; 2 Centre for Ecology, Evolution and Environmental Changes (cE3c), Faculdade de Ciências da Universidade de Lisboa, 1749-016 Lisboa, Portugal; 3 Biosystems & Integrative Sciences Institute (BioISI), Faculdade de Ciências da Universidade de Lisboa, 1749-016 Lisboa, Portugal; 4 Departamento de Biologia & CESAM, Universidade de Aveiro, 3810-193 Aveiro, Portugal; victor.bandeira@ua.pt (V.B.);

**Keywords:** Egyptian mongoose, gut microbiota, microbial profiling, bio-ecology, Mediterranean wild carnivores

## Abstract

The Egyptian mongoose (*Herpestes ichneumon*) is a medium-size carnivore that, in Europe, is restricted to Iberia. The bio-ecology of this species remains to be elucidated in several dimensions, including gut microbiota that is nowadays recognized as a fundamental component of mammals. In this work, we investigated the gut microbiota of this herpestid by single-molecule real-time sequencing of twenty paired male (*n* = 10) and female (*n* = 10) intestinal samples. This culture-independent approach enabled microbial profiling based on 16S rDNA and investigation of taxonomical and functional features. The core gut microbiome of the adult subpopulation was dominated by Firmicutes, Fusobacteria, Actinobacteria, and Proteobacteria. Eight genera were uniquely found in adults and five in non-adults. When comparing gut bacterial communities across sex, four genera were exclusive of females and six uniquely found in males. Despite these compositional distinctions, alpha- and beta-diversity analyses showed no statistically significant differences across sex or between adult and non-adult specimens. However, when function was inferred, males presented a significantly higher abundance of amino acid and citrate cycle metabolic pathways, compared to the significant overrepresentation in females of galactose metabolic pathways. Additionally, adults exhibited a significantly higher abundance of cationic antimicrobial peptide resistance pathways, while non-adults bared a significant overrepresentation of two-component systems associated with antibiotic synthesis, flagellin and biofilm production, and chemotaxis control. This study adds new insights into the mongoose bio-ecology palette, highlighting taxonomical and functional microbiome dissimilarities across sex and age classes, possibly related to primary production resources and life-history traits that impact on behavior and diet.

## 1. Introduction

The bioecology of each mammal species is a conjugation of different domains, such as geographic range and habitat, diet, genetics, morpho-physiology, social behavior and gut microbiota, the latter of which has been progressively acknowledged as a fundamental component of mammal biology [1]. The Egyptian mongoose (*Herpestes ichneumon* (Linnaeus, 1758)) is a carnivore species from the Herpestidae family with opportunistic feeding behavior and whose diet in the Mediterranean includes wild rabbit, other small mammals like rodents, reptiles, amphibians, birds, crayfish, eggs, and even carrion [2]. This species is mostly present in the African continent, but also in the Mediterranean Middle East, Turkey, and the Iberian Peninsula (Portugal and Spain) [3]. The historical process underlying Egyptian mongoose colonization of Iberia is an issue under debate. While Gaubert et al. (2011) support that mongooses reached Iberia through the Strait of Gibraltar during the Middle to Late Pleistocene sea-level fluctuations [4], more recently, Detry et al. (2018) suggested that this species might have been introduced by the Romans during their establishment in Hispania [5]. In Portugal, the species distribution in the early 20th century was restricted to south of the Tagus River [6], but it has gradually, and remarkably, expanded into central and north-eastern regions [7]. The drivers for this geographic expansion are subject to speculation but land-use changes in shrub-dominated ecosystems, deforestation, the transformation of agricultural practices, and climate change [8] seem to have jointly contributed to this phenomenon. The Egyptian mongoose has a home-range of about 3 km², inhabiting locals with scrub vegetation in coastal, lacustrine, and riparian habitats, avoiding humid forests and extreme deserts. In the Iberian Peninsula, it is found in Mediterranean maqui. Listed as of Least Concern, the species is widespread, common, and present in many protected areas. Ecological features of mongooses such as morpho- and stress-physiology, diet, body condition, or reproduction, have been unraveled in recent years [2], driven by the opportunity to explore a large array of specimen samples in Portugal, where it is a game species under the Portuguese hunting law [9].

In Portugal, both sexual and regional dimorphism in body size has been reported, attributed to different feeding behaviors across sex and regions, resulting in larger and heavier male adults in the south [10]. *H. ichneumon* exhibit variability in social organization, ranging from solitary individuals to groups, which show cooperative tendencies, particularly in areas with abundant food resources. The exclusive home-range use of males in high-density populations suggests the existence of a polygynous mating system, which is accomplished by the spatial distribution of females, in combination with the absence of paternal care behavior [11]. This species microbiota has been investigated through culture-dependent methods in two separate approaches: first, a preliminary study based on the limited bacteriological screening of 53 specimens [12], and the latter focused on the microbial characterization of the gut of 20 males and females using a broad range, systematic culturomics-like strategy [13]. This study enabled the isolation and characterization of a large array of aerobic and anaerobic bacterial microbiota, sporobiota, and mycobiota [13]. However, a deeper insight into comprehensive gut communities can only be accomplished by a culture-independent approach that complementarily allows the characterization of non-viable or viable but non-culturable bacteria. Third generation technologies have been revolutionizing genomic sequencing. Pacific Biosciences Single-Molecule Real-Time (PacBio SMRT) is one of these, producing an average read length of 10 kb to 15 kb (up to a maximum of 80 kb), with a throughput per run of 0.5 Gb to 1 Gb [14], which allows a more accurate taxonomical identification compared to previous sequencing techniques that generate much smaller reads.

In this work, we thus set out to explore the gut microbiota of Egyptian mongooses sampled in South Portugal using a phylogenetic marker gene sequencing approach based on the 16S rRNA gene. The aims of this study were to: (1) characterize the gut bacterial microbiota of the Egyptian mongoose population; (2) investigate sex and age class-related taxonomic and functional differences; (3) identify statistically significant associations between microbiota and biotic and/or abiotic factors. 

## 2. Materials and Methods

### 2.1. Egyptian Mongoose Specimens

Egyptian mongoose carcasses (ten male and ten female) obtained from legal predator density control actions were opportunistically used for this work. These carcasses were donated by hunters for scientific purposes and, after death, were frozen at −20 °C until necropsy. No animals were sacrificed for this study. The twenty animals under analysis were selected from a wider array of available mongooses based on several biological factors, namely sex, age class, geographic location, land-use and stomach content at the time of death. They were harvested from the Baixo Alentejo region, south of the Tagus River, from a landscape predominated by agroforestry and agriculture. The selected animals had the same stomach content at death, mostly composed of mammal and egg items [2]. Age class distribution was 16 adults and four non-adults (two subadults and two juveniles). 

Mongooses were subjected to necropsy and specimen collection by pathologists at the necropsy facilities of the National Institute for Agrarian and Veterinary Research (INIAV, IP), the National Reference Laboratory for Animal Health. No signs of putrefaction or disease were detected. The abdominal cavity of each specimen was opened and the intestines isolated. Solid intestinal content (colon) was collected from each animal using a sterile feces collection tube and immediately processed for further analysis.

### 2.2. DNA Extraction, Quantification, Sequencing and Reads Processing

DNA was extracted from 500 mg of feces from each mongoose using the NZYSoil gDNA isolation kit (NZYTech, Lisbon, Portugal) following the manufacturer’s instructions. DNA was quantified using a Qubit fluorometer (Qiagen, Venlo, Netherlands), following the manufacturer’s instructions. The full-length 16S rRNA gene was amplified from 2.5 ng/µL of total DNA from each intestinal sample, using the universal primers 27F (5’-AGAGTTTGATCCTGGCTCAG-3′) and BS-R1407 (5′-GACGGGCGGTGWGTRC-3′), resulting in the amplification of variable regions (V) V1 to V8, originating an expected size amplicon of 1381 bp [15]. These amplicons were commercially sequenced on the PacBio SMRT RS-II platform (Pacific Biosciences, CA, USA; commercially available at Eurofins Genomics, Germany). The more recent PacBio P6-C4 chemistry was used.

Data were preprocessed using the PacBio SMRT Analysis Portal (Pacific Biosciences, CA, USA), generating single-molecule circular consensus sequences (CCS). The resulting CCS were analyzed using the EzBioCloud platform [16] (Seoul, Republic of Korea; accessed on January 2019). The PKSSU4.0 taxonomy database of prokaryotic 16S rRNA gene sequences was used. The uploaded CCS were trimmed of primers and quality filtered under several criteria, namely sequence length out of the expected range (<80 bp or >2000 bp), sequences with an average Q value lower than 25, sequences not predicted as being from the 16S rRNA gene by the Hidden Markov Model, and sequences found to be singletons when clustering using a cutoff of 97% similarity by the UCLUST method [17]. After, the pool of CCS reads was denoised using the Discrete Universal Denoiser (DUDE)-Seq software [18] and non-redundant reads were extracted. Next, the resulting reads were taxonomically assigned using USEARCH [17]. The following similarity cutoff values were used for taxonomical identification: species (≥97%), genus (between 96.9% and 94.5%), family (between 94.4% and 86.5%), order (between 86.4% and 82%), class (between 81.9% and 78.5%), and phylum (between 78.4% and 75%) [19]. Following, the non-assigned sequences were subjected to the UCHIME program [20] for the detection of chimerical sequences.

The valid reads (all reads that passed the previous filters) were then used for Operational Taxonomic Unit (OTU) picking, using an “open-reference” method with the following steps: (1) species-level identification clustering using the taxonomic assigned data; (2) OTU clustering using the UCLUST tool; and (3) a conjugation of the clusters obtained by the two previous steps. Singletons were omitted from further analysis. Also, the Good’s coverage of library was calculated to assess the representativity of the obtained reads when compared with the actual population [21].

### 2.3. Estimation of Alpha-Diversity Indices

Using OTU information, several diversity indices were estimated to measure bacterial species richness (Abundance-based Coverage Estimator (ACE) [22], Chao1 [23], Jackknife [24], and number of OTU) and evenness (Shannon [25], Simpson [25], and non-parametric Shannon (NPShannon) [26]), using the EzBioCloud platform. Besides these estimators, rarefaction and rank abundance curves were also plotted [27,28].

### 2.4. Comparison between Subpopulations Using Beta-Diversity Indices

The microbial communities at the genus level of each individual host were grouped according to host biological features, namely host sex (male and female) and age (adult and non-adult). Next, the subpopulations were compared using both ordination analysis and hierarchical clustering. First, a distance matrix was calculated using four possible metrics: UniFrac [29], Generalized UniFrac [30], Bray-Curtis [31], and Jensen-Shannon [32]. Then, the resulting matrix was used to compute a principal coordinate analysis (PCoA) or cluster using the Unweighted Pair Group Method with Arithmetic Mean (UPGMA) algorithm, originating a dendrogram. The beta-diversity analysis was also performed using the EzBioCloud platform.

### 2.5. Taxonomic and Functional Biomarker Discovery

To assess the taxonomic biomarkers specifically found in each host subpopulation, multiple comparisons between the different taxa were performed using a Kruskal-Wallis H test (α = 0.05) coupled with a Benjamini-Hochberg correction test. To infer and predict the differential functional profile of the host subpopulations, the Phylogenetic Investigation of Communities by Reconstruction of Unobserved States (PICRUSt) algorithm was used, using an up-to-date taxonomic database of 16S rRNA genes, species genomes, and the Kyoto Encyclopedia of Genes and Genomes (KEGG) information as the input. This algorithm predicts which gene families are present in the sample using 16S rRNA data and estimates the overall metagenome composition and function [33]. The obtained functional profile is compared between host subpopulations using a linear discriminant analysis effect size (LEfSe) (α = 0.05) [34]. Both analyses were performed using the EzBioCloud platform.

### 2.6. Abiotic and Biotic Data Integration

For microbiota and bio-environmental data integration, we performed a Principal Component Analysis (PCA) using available information for the 20 Egyptian mongoose specimens [2,10]. The abiotic variables used were related to land-use, climatic data, road net, river net, and population data. The biotic variables used were related to stomach content at the time of death and different body measurements. Both biotic and abiotic data available were coupled with the gut microbiota genera detected and a PCA was calculated, enabling the prediction of the most influential biotic and abiotic variables to the gut microbiota composition of the Egyptian mongoose. Data integration was performed using the R software (Version 3.6.1, Vienna, Austria).

### 2.7. Data Analysis

Considering alpha-diversity indices, results from indices estimations were displayed as means of values of the individuals belonging to each subpopulation with respective standard deviation. When comparing two conditions, the Wilcoxon rank-sum test (α = 0.05) was performed. Considering beta-diversity, comparison of microbial communities between subpopulations of hosts were evaluated using beta set-significance analysis, namely a permutational multivariate analysis of variance (PERMANOVA) test. Both statistical analyses were performed using the EzBioCloud platform.

## 3. Results

### 3.1. Analysis of the Sequencing Data

A total of 46,508 reads across all samples were generated through SMRT sequencing. The trimming based on quality detected 1091 low-quality amplicons. Next, the clustering and taxonomic assignment detected seven non-target amplicons. Also, the chimera search detected 4187 chimerical amplicons. So, the total number of valid reads was 41,223 (88.6%). The average length of these reads was approximately 1416.6 (± 10.8) nucleotides, with an average minimum length of 1320 (± 10.53) nucleotides and an average maximum length of 1478 (± 26.86) nucleotides. A total of 37,565 (91.1%) sequences were identified at a species level. Together, this work accomplished a Good’s coverage of library of 99.18% (±0.46). For more information regarding individual sample data, see Supplementary Table S1.

### 3.2. Bacterial Composition of the Egyptian Mongoose Gut on a Population and Individual Level

**Figure 1:**
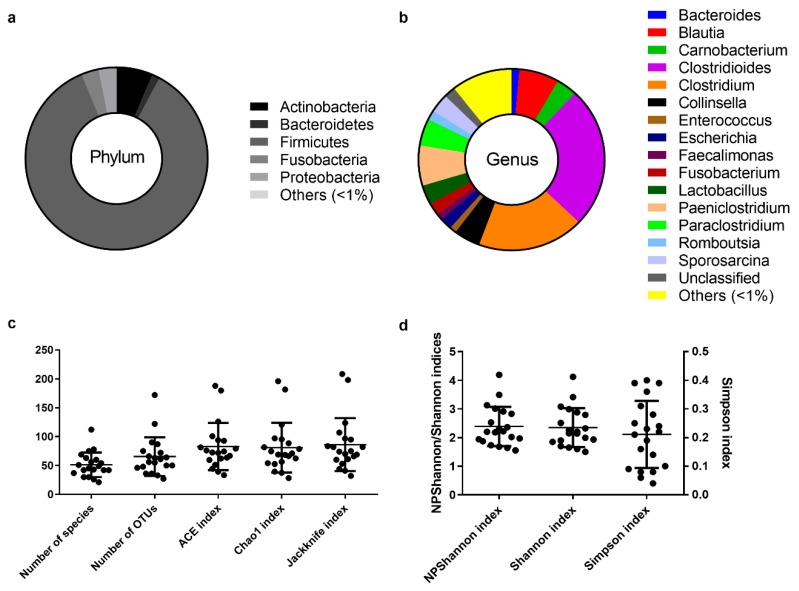
The core intestinal bacterial microbiota of the Egyptian mongoose population. (**a**) Relative abundance on a phylum and (**b**) genus level. (**c**) Species richness (number of species, number of OTUs, ACE, Chao1, and Jackknife indices) and (**d**) species evenness (NPShannon, Shannon, and Simpson indices).

The assignment of consensus taxonomy resulted in the identification of 11 phyla being represented across the intestinal samples of the Egyptian mongoose population. On a populational level, all intestinal samples were dominated by Firmicutes (86%), followed by Actinobacteria (6%), Fusobacteria (3%), Proteobacteria (3%), and Bacteroidetes (1%) (Figure 1a). Besides these phyla, some rare phyla were also detected (0.04%), namely Chloroflexi, Cyanobacteria, Planctomycetes, Saccharibacteria, TM6, and Verrucomicrobia. 

The initial genus-level analysis focused on OTUs with an abundance greater than 1%. Genus level OTU classification resulted in the identification of 16 genera present in the gastrointestinal tract of Egyptian mongooses, with a relative abundance greater than 1% in any given sample (Figure 1b). *Clostridioides* (25%) and *Clostridium* (19%) were the most abundant genera, representing almost half of the bacterial population (44%). At a lower, but still high, abundance, *Blautia* (7%) and *Paniclostridium* (7%) genera were identified. Besides these genera, *Collinsella* (5%), *Paraclostridium* (5%), *Carnobacterium* (4%), *Lactobacillus* (3%), *Sporosarcina* (3%), *Escherichia* (2%), *Fusobacterium* (2%), *Romboutsia* (2%), *Bacteroides* (1%), *Enterococcus* (1%), and *Faecalimonas* (1%) were also detected. A total of 10% of reads were assigned to genera present in <1% relative abundance and only a total of 2% of the reads were not assigned to a genus.

**Figure 2:**
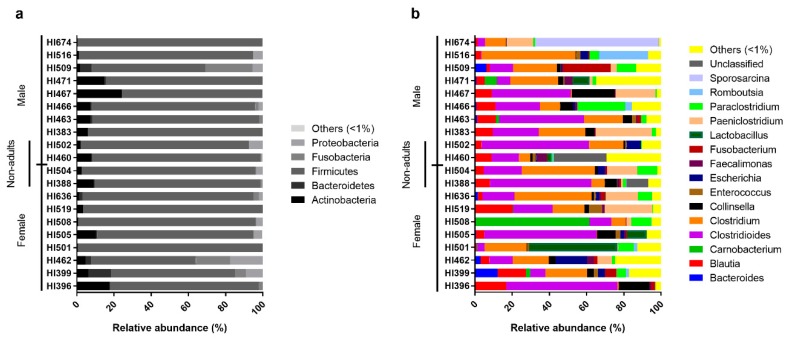
Relative abundance of the individual intestinal bacterial microbiota of the Egyptian mongoose on a phylum (**a**) and genus (**b**) level.

On an individual level, Egyptian mongoose specimens exhibited similar microbial profiles, with Firmicutes prevailing (Figure 2a). However, some notorious individual differences could be highlighted in relation to the overall population, namely mongoose individual HI467 that holds a higher abundance of Actinobacteria and mongoose HI509 that presents a higher abundance of Fusobacteria. Regarding genera compositions (Figure 2b), a higher dissimilarity among individuals could be detected, with a more pronounced difference in mongoose HI674 which possessed a higher abundance of *Sporosarcina* sp. (66%); HI508 with a higher abundance of *Carnobacteria* sp. (61%); HI501 with a higher abundance of *Lactobacillus* sp. (47%); HI516 with a higher abundance of *Romboutsia* sp. (26%); and HI466 with a higher abundance of *Paraclostridium* sp. (25%). Mongoose HI460 shows a higher abundance of unclassified reads (28%) at the genus level.

**Figure 3:**
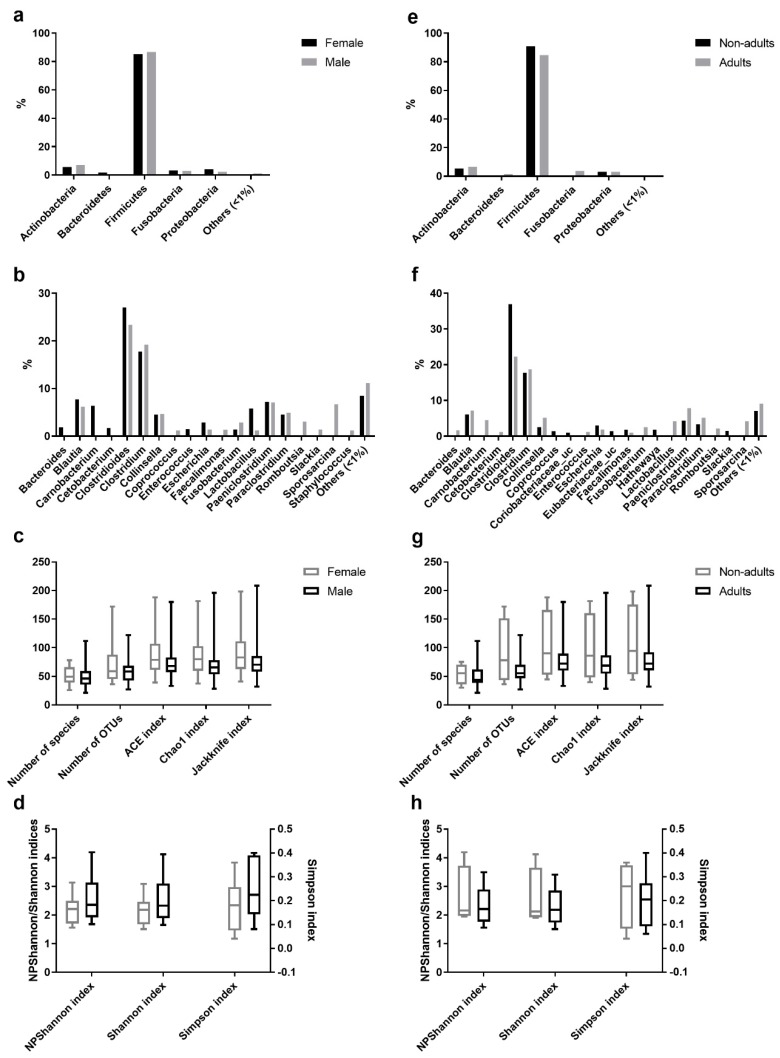
Comparison of the intestinal bacterial microbiota of the Egyptian mongoose between sex and age class. (**a**) Relative abundance on a phylum and (**b**) genus level of female and male individuals. (**c**) Species richness (number of species, number of OTUs, ACE, Chao1, and Jackknife indices) and (**d**) species evenness (NPShannon, Shannon, and Simpson indices) of female and male individuals. (**e**) Relative abundance on a phylum and (**f**) genus level of adult and non-adult individuals. (**g**) Species richness (number of species, number of OTUs, ACE, Chao1, and Jackknife indices) and (**h**) species evenness (NPShannon, Shannon, and Simpson indices) of adult and non-adult individuals.

Comparative analysis of OTUs across male and female intestinal samples evidenced that the majority of OTUs were shared across the individuals (Figure 3). Of the 174 different genera identified in the gastrointestinal tract of Egyptian mongoose individuals (including OTUs with <1% relative abundance), 19 were present in >1% relative abundance in at least one subpopulation (male or female); nine were found to be shared between sexes, four were found to be unique to the female hosts (*Bacteroides*, *Carnobacterium*, *Cetobacterium*, and *Enterococcus*), and six unique to the male hosts (*Coprococcus*, *Faecalimonas*, *Romboutsia*, *Slackia*, *Sporosarcina*, and *Staphylococcus*) (Figure 3b). Apart from these dissimilarities, similar proportions of phyla and genera were observed, with Bacteroidetes being uniquely found in the gastrointestinal tract of females (Figure 3a). However, these differences were statistically non-significant.

A comparative analysis of OTUs identified in the adult and non-adult intestinal samples found that the majority of OTUs were shared across individuals (Figure 3). Of the 174 different genera identified in the gastrointestinal tract of Egyptian mongoose individuals (including OTUs with <1% relative abundance), 21 were present in >1% relative abundance in at least one subpopulation (adult or non-adult); eight were shared between age classes; eight were unique to the adult hosts (*Bacteroides*, *Carnobacterium*, *Cetobacterium*, *Enterococcus*, *Fusobacterium*, *Lactobacillus*, *Romboutsia*, and *Sporosarcina*), and five unique to the non-adult hosts (*Coprococcus*, *Coriobacteriaceae*_uc, *Eubacterium*_uc, *Hathewaya*, and *Slackia*) (Figure 3f). Besides, similar proportions of phyla and genera were observed. However, Bacteroidetes and Fusobacteria were uniquely found in the gastrointestinal tract of adults (Figure 3e). Furthermore, these differences were statistically non-significant.

### 3.3. Estimation of Alpha Diversity Indices

Among the species richness indices, ACE, Chao1, Jackknife, and the number of OTUs were estimated. ACE and Chao1 are only sensitive indicators to rare OTUs [22,23], and Jackknife is sensitive to abundant OTUs [24], with higher values indicating higher diversity. Besides the difference in sensitivity, all species richness indicators showed similar values for all fecal samples (Figure 1c) and no significant differences across sex (Figure 3c) and age (Figure 3g) were found, suggesting low variability across this Egyptian mongoose population sample.

Among the species evenness indices, Shannon, Simpson, and NPShannon, were estimated. The Shannon and Simpson indicators are the most commonly used in microbial community studies, with the first displaying values higher than zero, and the maximum value corresponding to an equal distribution among all species present [25]. The second displays the probability of two randomly selected sequences belonging to the same species and uses this as a *proxy* to define even populations to the ones showing lower values [25]. NPShanon is a non-parametric estimation of the Shannon index and takes into consideration undetectable species and species of unknown abundance in a given sample [26]. Besides individual differences in considered variables, all species evenness indicators showed similar values across all fecal samples (Figure 1d). No significant differences across sex (Figure 3d) and age (Figure 3h) were found, suggesting, once again, limited variability across this Egyptian mongoose subpopulation.

Besides these estimators, rarefaction and rank abundance curves were also plotted. The rarefaction curve is the correlation between the number of reads and the number of OTUs and the steeper the slope, the higher the diversity [27]. Rarefaction curves show a good depth of sequence coverage, with curves beginning to level off after 1500 reads (Supplementary Figure S1). The rank abundance curve is the correlation between the rank of OTUs and the relative abundance of OTUs at each rank—the steeper the slope, the lower the diversity [28]. Rank abundance curves obtained from the OTU list evidence low species evenness across all fecal samples, indicated by the relatively steep curve, highlighting a greater abundance of high-ranking species comparing to low-ranking ones (Supplementary Figure S2).

### 3.4. Comparison between Subpopulations Using Beta-Diversity Analysis

The beta-diversity analysis helps to assess the relationship of microbial communities of different subpopulations. This can be performed using different metrics to calculate the dissimilarity/distance matrix, such as UniFrac, Generalized UniFrac, Bray-Curtis, and Jensen-Shannon. UniFrac is a distance measure obtained from the comparison between microbial communities based on the phylogenetic analysis of their OTU [29]. Generalized UniFrac distance unifies the weighted and unweighted UniFrac, correcting their limitations by decreasing the importance given to either abundant or rare OTUs, respectively [30]. The Bray-Curtis dissimilarity measure is obtained by comparing the counts of each OTU in different communities [31]. The Jensen-Shannon distance is the square root of the Jensen–Shannon divergence and is based on the comparison of the probability distribution of two microbial communities [32]. These dissimilarity/distance matrices can be used in an ordination analysis (such as Principal Coordination Analysis (PCoA)) or clustering in a hierarchical analysis. 

Besides numerical differences in the PCoA obtained from the different metrics, samples clustered in a similar way, with samples clustering throughout the plot and with no clear differentiation between host sex (Supplementary Figure S3) or host age-class (Supplementary Figure S4), suggesting a comparable phylogenetic diversity across all fecal samples on a bacterial genus level. However, the fecal samples from non-adult individuals seem to be plotted in close proximity, suggesting a similar phylogenetic diversity. The hierarchical analysis using the UPGMA agglomeration metric shows similar dendrograms between UniFrac and Generalized UniFrac (Supplementary Figures S3c,f and S4c,f), and between Bray-Curtis and Jensen-Shannon (Supplementary Figures S3i,l and S4i,l), but they were different between them. The observed clusters do not group intestinal samples according to host sex or age class, reinforcing the idea of a comparable phylogenetic diversity. PERMANOVA results show no significant difference between bacterial gut microbiota of male and female hosts and between non-adult and adult hosts (Supplementary Table S2).

### 3.5. Taxonomic Biomarker Discovery

To assess the taxonomic biomarkers of male, female, adults, and non-adults, a Kruskal-Wallis H test was performed. Regarding sex (Supplementary Table S3), only two genera and three species were significantly more abundant (*p*-value = 0.03) in male mongooses when compared to female mongooses, and one species was significantly more abundant (*p*-value = 0.03) in female mongooses when compared to male. Regarding age class (Supplementary Table S4), one class (*p*-value = 0.034), two orders (*p*-value = 0.034 and 0.048), six families (*p*-value = 0.003 to 0.054), 24 genera (*p*-value = 0.004 to 0.051), and 53 species (*p*-value = 0.004 to 0.055) were significantly more abundant in non-adult mongooses when compared with adults.

### 3.6. Functional Biomarker Discovery

To predict the differential functional profile of female, male, adults, and non-adults, a LEfSe analysis was performed. LEfSe identifies units that are highly associated with a group in a dataset, using a non-parametric statistical test and estimating a size effect score for each differentially abundant feature using linear discriminant analysis [34]. The functional analysis can be based on three KEGG categories: orthology, module, or pathway [35]. Regarding sex (Supplementary Table S5), 23 orthologs (*p*-value = 0.007 to 0.051), two modules (*p*-value = 0.029 and 0.035), and three pathways (*p*-value = 0.037 to 0.054) were significantly different in abundance, with seven orthologs and one pathway being significantly more abundant in samples of female individuals, and 16 orthologs, two modules, and two pathways being significantly more abundant in samples of male individuals. Regarding age class (Supplementary Table S6), 19 orthologs (*p*-value = 0.006 to 0.049), one module (*p*-value = 0.038), and one pathway (*p*-value = 0.048) were significantly different in abundance, with four orthologs and one module being significantly more abundant in samples of adult individuals, and 15 orthologs and one pathway being significantly more abundant in samples of non-adult individuals.

### 3.7. Abiotic and Biotic Data Integration

**Figure 4:**
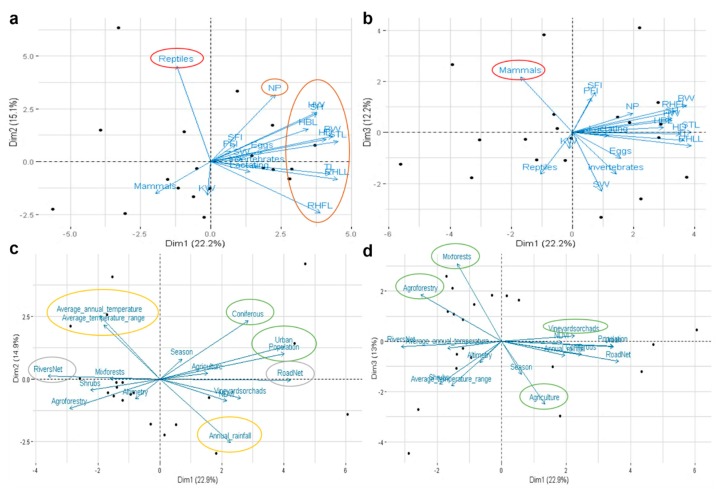
Interaction between bacterial gut microbiota and biotic and abiotic factors of Egyptian mongoose. Principal Component Analysis using: (**a**,**b**) biological variables related to stomach content at the time of death and different body measurements; (**c**,**d**) environmental variables related to land-use, and climatic, topographic, and population data. The more influential factors are delimited using different colors—red: stomach content; brown: biometric data; green: land-use; yellow: climatic data; grey: topographic data. BW: body weight; STL: snout-tail length; TL: tail length; HBL: head and body length; RHLL: right hind leg length; RHFL: right hind foot length; SH: shoulder height; NP: neck perimeter; HD: head diameter; HW: head width; SW: spleen weight; KW: kidney weight; SFI: solid fat index; PFI: perivisceral fat index.

For microbiota and bio-environmental data integration, we performed a Principal Component Analysis integrating 35 variables, including 19 biotic factors, with the microbiota data. Reptile and mammal stomach contents and all biometric factors tested (body weight (BW), snout-tail length (STL), tail length (TL), head and body length (HBL), right hind leg length (RHLL), right hind foot length (RHFL), shoulder height (SH), neck perimeter (NP), head diameter (HD), head width (HW), spleen weight (SW), kidney weight (KW), solid fat index (SFI), and perivisceral fat index (PFI)) were more influential on the bacterial microbiota in the Egyptian mongoose gut (Figure 4a,b). Additionally, several abiotic factors were also tested (*n* = 16), with land-use, temperature, rainfall, river net, and road net being the more influential on the gut microbiota of the Egyptian mongoose (Figure 4c,d).

## 4. Discussion

Gut microbiota is nowadays an interdisciplinary and central research topic due to its recognized importance in shaping mammalian biology. In this study, we generated an extended sequence library of the gut microbiota of the Egyptian mongoose, highlighting sex and age class-related differences on taxonomic and functional levels. To accomplish this level of information on the gut microbiome of this carnivore species, a culture-independent approach was used to complement the first insights generated by previous culturomic-based studies [12,13]. The use of PacBio SMRT technology enabled the production of single long reads with the full extension of the 16S rRNA gene sequence, leading to a more reliable taxonomic identification of bacterial communities with high resolution, sensitivity, and accuracy. This phylogenetic marker gene strategy reinforced the notion that the gut microbiota of the Egyptian mongoose adult population is remarkably dominated by Firmicutes (*Blautia*, *Clostridioides*, *Clostridium*, and *Lactobacillus*), followed, in markedly lower proportions (in decreasing order), by Actinobacteria (*Collinsella*), Fusobacteria (*Fusobacterium*), and Proteobacteria (*Escherichia*). 

Firmicutes are one of the most abundant bacterial phyla in the mammalian gastrointestinal tract, assuring protein degradation, the preservation of gut homeostasis and host immunity development [36]. Within Firmicutes, Clostridia members represented 66% of the bacterial gut microbiota of the Egyptian mongoose. Their ability to breakdown carbohydrates and proteins and to promote nutrient absorption [37] place these proteolytic bacteria as central within mammal microbiota [38] under the presence of high-protein content diet [37,39]. Detection of these and other bacterial phyla in our study is consistent with the carnivorous diet of the Egyptian mongoose [10,40]. The Bacilli members, like *Lactobacillus* spp., which are also reported in the gut of vertebrates [41], possessing prebiotic and probiotic activities [42], were also found in the mongoose gut.

Interestingly, the Actinobacteria phylum members that are usually a minor fraction of the gut microbiota of mammals [36] were the second most represented phyla in this study. *Collinsella*, previously correlated with human lipid metabolism [43], was the most prevalent genus within that phylum and is probably related to a high-fat diet, reinforcing, once again, the consistency of our findings with the carnivorous dietary patterns of the Egyptian mongoose.

Also in agreement was the detection of the Fusobacteria phylum, particularly *Fusobacterium* spp. that ferment carbohydrates and amino acids to produce a variety of organic acids, such as acetic acid, formic acid, butyric acid [36] and short-chain fatty acids, which account for host energy sources and regulate the metabolism of glucose, cholesterol, and fatty acids [44].

Proteobacteria members, particularly Enterobacteriaceae such as *Escherichia* spp., were also found. These are common commensals in mammals, with extremely diverse metabolism that includes the ability to break down and ferment complex sugars and produce vitamins [36]. They have been reported as predominant in other Carnivora members, such as grizzly bears and giant pandas [37,45,46]. A high ratio of Proteobacteria/Bacteroidetes (calculated ratio of 3) was evident in the Egyptian mongoose samples surveyed. This finding has been previously related with a carnivorous or scavenger diet, namely in carnivores like cheetah, Tasmanian devil, spotted hyena, and polar bear [47], and also with a very efficient harvest of energy [48].

A previous study by our group using culture-dependent methods followed by 16sRNA gene sequencing of selected isolates was performed upon the same 20 Egyptian mongoose specimens surveyed in this work [13]. The detection of the phyla Firmicutes (67%), Proteobacteria (32%), and Actinobacteria (1%) was registered, enclosing twenty genera. Strikingly, the *Delftia*, *Ralstonia*, *Rummelibacillus*, *Stenotrophomonas*, *Pantoea*, *Solibacillus*, and *Robinsoniella* genera were exclusively found when using a culture-dependent approach. The disparities between the data generated by both methods can be caused by the length of the 16S rRNA gene sequence used to identify the bacterial isolates in the former study, which sometimes were of shorter length, possibly leading to taxonomic misidentification, allied with the strong bias that can be associated with culture-dependent methods performed on limited cultivability conditions (limited number of growth media, oxygenation, temperature, among others). However, culture-independent approaches have a minimum sequence concentration threshold, which could explain the lack of detection of poorly represented OTUs. This limitation can be overcome by culture-dependent and taxonomical enrichment approaches [49]. Discrepancies between culture-dependent and culture-independent methodologies are frequently reported by others [50,51,52,53].

Regarding sex-related differences, the male-specific bacterial groups detected here were the *Faecalimonas* genus that is usually found in the mammalian gut [54,55], the *Romboutsia* genus that is normally found in humans and rats [56,57], and the *Sporosarcina* genus typically found in birds [58]. Female-specific taxa comprised *Carnobacterium*, *Enterococcus,* and *Cetobacterium* genera, together with Bacteroidetes. The first genus was previously detected in food (fish, meat, and some dairy products) and natural environments (sediments and water) [59]; however, to our knowledge, *Carnobacterium* spp. has only been identified in the gastrointestinal microbiota of the Egyptian mongoose both by sequence-dependent and -independent methods [12,13]. Detection of Enterococci is in agreement with other works on wild animals conducted in Portugal, particularly carnivores [13,60,61,62]. The *Cetobacterium* genus is commonly found in the intestines of freshwater fish species [63,64,65], humans [66], and dog feces [67]. This genus has the ability to ferment peptides and carbohydrates and to produce vitamin B12 that can be absorbed by the host [65,66]. Bacteroidetes are cosmopolitan bacteria, being one of the most frequently found members in mammalian gastrointestinal microbiota [36]. These bacteria have the ability to degrade proteins and carbohydrates. The fermentation of these compounds releases short-chain fatty acids [36,68]. Also, they can interact with the immune system of the host, activating T-cells and protecting the gastrointestinal tract from pathogenic bacteria [69], although some sporadic reports of opportunistic infections are also available [70].

In our previous culture-dependent study [13], the microbial load of intestinal samples seeded in a rich medium under anaerobiosis was higher in females than in males, as indicated by aerobic/anaerobic vegetative and sporobiota communities. Also, *Paeniclostridium* spp., *Pantoea* spp., *Sporosarcina* spp., *Solibacillus* spp., and *Stenotrophomonas* spp. isolation was restricted to female individuals, while *Paenibacillus* spp., *Propionibacterium* spp., *Robinsoniella* spp. and *Staphylococcus* spp. were only isolated from males.

Regarding the taxonomic biomarker discovery analysis, we detected a significantly higher abundance of *Kocuria* spp., *Hathewaya* spp., and *Clostridium haemolytium* in male samples. The *Kocuria* genus is a typical initial gut colonizer and is normally found in the intestine, but more typically present on other mucosae of mammals [71]. *Clostridium haemolytium* is the causative agent of bacillary hemoglobinuria, mostly occurring in cattle [72]. Female samples showed a significantly higher abundance of *Clostridium mediterraneense*, a recently discovered species isolated from the human gut in France [73]. Besides these compositional differences, the beta-diversity analysis showed no statistically significant differences across sex. Compositional sex differences in gut microbiota could be the result of dietary, behavioral and/or host physiology distinctions. In fact, previous studies exploring biometric, diet and splenic data of Egyptian mongooses from the same biogeographic region found dietary and immune system differences across sex [2,74]. Other studies in primates have also shown sex-specific bacterial microbiota frameworks [75,76,77].

Regarding age-related differences, several main bacterial groups were absent from non-adults, while the *Coprococcus* genus was specifically associated with this age class, as was *Slackia* spp. previously found in gut samples from porcupine, beaver, coyote, and Arctic wolf [78]. Besides the latter, other members of Coriobacteriaceae are common mammalian symbionts, responsible for the conversion of bile salts and steroids and activation of polyphenols [79]. Moreover, *Eubacterium* and *Hathewaya* genera were also confined to non-adult specimens. The presence of these species can indicate an increased digestive and absorptive capacity, promoting an increase in weight and size, which is essential for the normal development of juveniles and an increase in the immune system capacity when reaching the adult period due to an increase of vitamin production and the interaction of bacterial-epithelial cells in the gastrointestinal tract.

Reinforcing these notions, the taxonomic biomarker discovery analysis detected a significantly higher abundance of Tissierellia and Clostridia classes, Erysipelotrichales order, Eubacteriaceae, Cellullomonadaceae, Rikenellaceae, and Peptoniphilaceae families in non-adult samples. Tissierellia members have been previously detected in the gut microbiota of Canadian mink [80] and humans [81,82]. Erysipelotrichales members were associated with lipidemic profiles of human hosts, probably associated with high-fat content diets [83], together with Rikenellaceae members [84]. Eubacteriaceae bacteria have been reported as major members of the gut microbiota of Forest Musk deer [85]. Cellulomonadaceae are considered probiotic species that can convert cellulose into other metabolites [86]. Peptoniphilaceae were reported in the gut microbiota of children [81] and women [87]. Besides compositional differences, the beta-diversity analysis also showed no statistically significant differences across age classes of the Egyptian mongoose. In contrast, results from the culture-dependent methodology [13] evidenced similarities between adults and juveniles, with sub-adults clustering separately. This common framework for adults and cubs was attributed to the social behavior of the species which relies on the protection and feeding of the cubs, scent marking and social latrines [11]. This higher proximity and interaction between adults and juveniles can increase diet similarity and fecal-oral transmission of microbiota [11]. This phenomenon was previously reported in mice, birds, and humans [55].

For the first time, the differential functional profile of the Egyptian mongoose population was evaluated. Male Egyptian mongooses showed a significantly higher abundance of catabolic pathways of valine, leucine, and isoleucine amino acids. This degradation is usually performed by members of the Clostridia class [88], such as *Coprococcus*, *Faecalimonas*, and *Romboutsia* genera, which are more abundant in male gut microbiota. The male microbiota also had a significantly higher abundance of tryptophan metabolism pathways. This metabolic route is usually conducted by *Peptostreptococcus* spp., *Lactobacillus* spp., and *Clostridium* spp. [89]. Male individuals did have an overrepresentation of closely related members of *Peptostreptococcus* (i.e., *Romboutsia*). In contrast, the female microbiota showed an overrepresentation of closely related members of *Lactobacillus* (i.e., *Carnobacterium* and *Enterococcus*). Male hosts also revealed a significantly higher abundance of citrate cycle modules in their microbiota. The citrate cycle includes several amino acid metabolites, such as valine, leucine, isoleucine, and tryptophan, all of which have differentially functioning synthesis in the bacterial community of the male gut. Female mongooses exhibited a significantly higher abundance of galactose metabolic pathways, normally performed by *Bacteroides* (in particular, *Bacteroides vulgaris* [90]), which is usually overrepresented in female hosts. These findings suggest that the bacterial gut microbiome of the Egyptian mongoose is modulated by sex-specific strategies to produce energy.

In adult Egyptian mongooses, cationic antimicrobial peptide (CAMP) resistance genes were significantly overrepresented, specifically the dltABCD operon of Gram-positive bacteria. CAMP are short peptides secreted by immune and epithelial cells in response to bacterial products, such as lipopolysaccharide (LPS) or other inflammatory signals [91]. The resistance to CAMP enables bacterial virulence and resistance to innate immunity mechanisms, increasing immune evasion [91]. This finding may indicate the presence of opportunistic pathogenic bacteria in the bacterial community of the adult gut, even though no signs of disease could be perceived during necropsy. Also, no high percentage of opportunistic pathogenic Gram-positive bacteria could be found. In non-adult Egyptian mongooses, two-component systems associated with antibiotic synthesis, flagellin production, chemotaxis control, and biofilm formation genes were significantly overrepresented. All these processes are related to bacterial virulence and pathogenesis, which may indicate an overrepresentation of opportunistic bacteria in the gut of juvenile Egyptian mongooses, possibly enabled by an immature immune system, even though signs of disease were not perceived.

Abiotic and biotic factors such as land-use, climatic and topological data, feeding, and biometric data were found to exert an influence in the bacterial gut of the Egyptian mongoose. The effect exerted by abiotic factors on mammalian microbiota, such as alterations in native habitat, has been reported by others [92]. Shifts in land-use and topography that cause shifts in food availability, quality, or composition have been described to impact the gut microbiome [93,94,95]. Temperature and rainfall can affect bacterial microbiota by directly altering environmental microbial communities and indirectly by inducing changes in primary production and host physiology [96,97,98]. Biotic factors such as host biometry can modify bacterial abundance and diversity, being associated with host health [95]. Other intrinsic host-associated factors, such as diet, and extrinsic features, such as land-use and climatic changes, can both lead to gut microbiota adaptations as a result of the Egyptian mongoose expanding its range across environmental gradients [8].

## 5. Conclusions

This study represents the first description of the Egyptian mongoose gastrointestinal microbiota using culture-independent methods, improving our knowledge on the bioecology of this species. It leads to a better understanding of an until now poorly characterized carnivore, together with a sex and age class comparative analyses, that help to assess the indirect effects exerted by host behavior, diet, reproduction, and other biological characteristics on gut microbiota composition and function. Altogether, our results also reinforce the need to use a combination of both culture-dependent and culture-independent strategies when the aim is to capture the maximum biodiversity across complex samples. Finally, our efforts emphasize the value of microbiome studies to fully comprehend mammalian species ecology in light of behavior, diet, and geographic features.

## Supplementary Materials

The following are available online at https://www.mdpi.com/2076-2607/8/3/392/s1, Table S1: Data of read analysis for the 20 intestinal samples of Egyptian mongoose specimens, Table S2: PERMANOVA test results of the microbial community of Egyptian mongoose comparison between female and male and between non-adult and adult, Table S3: Taxonomic biomarkers discovery of female and male Egyptian mongoose fecal bacterial microbiota using a Kruskal-Wallis H test, Table S4: Taxonomic biomarkers discovery of non-adult and adult Egyptian mongoose fecal bacterial microbiota using a Kruskal-Wallis H test, Table S5: Functional biomarkers discovery of female and male Egyptian mongoose fecal bacterial microbiota applying a LEfSe analysis, Table S6: Functional biomarkers discovery of non-adult and adult Egyptian mongoose fecal bacterial microbiota applying a LEfSe analysis, Figure S1: Rarefaction curves for the twenty different fecal samples of Egyptian mongoose measured individuals using the observed species metric, Figure S2: Rank abundance curves for the twenty different fecal samples of Egyptian mongoose individuals using the observed species metric, Figure S3: Beta-diversity analysis to compare the fecal bacterial microbiota, on a genus level, of male and female Egyptian mongoose, Figure S4: Beta-diversity analysis to compare the fecal bacterial microbiota, on a genus level, of adult and non-adult Egyptian mongoose.
