# Supplementary material for: Egyptian Mongoose (Herpestes ichneumon) Gut Microbiota: Taxonomical and Functional Differences across Sex and Age Classes
Source: Microorganisms. 2020 Mar 11;8(3):392. doi: 10.3390/microorganisms8030392 (PMC7143146; doi:10.3390/microorganisms8030392)
Supplement: Supplementary file 1 [file microorganisms-08-00392-s001.pdf]

Supplementary Table S1 – Data of read analyses for all 20 fecal samples of the Egyptian mongoose

ID	Total reads	Low-quality amplicons	No-target amplicons	Chimeric amplicons	Min length	Average length	Max length	Valid reads	Number of reads ID at the species level	Good's coverage of library (%)
383	2083	33	0	281	1302	1407.0	1442	1769	1722	99.72
466	2373	50	1	212	1310	1409.2	1478	2110	1882	99.53
467	1856	53	3	187	1308	1404.2	1453	1613	1555	99.19
516	2397	36	0	147	1316	1412.2	1476	2214	2161	99.10
460	2657	297	0	246	1302	1416.4	1485	2114	1169	98.77
463	2023	34	0	189	1339	1411.4	1561	1800	1677	99.44
471	2290	41	0	359	1325	1430.1	1490	1890	1833	97.57
502	2565	31	0	227	1315	1411.4	1481	2307	2240	99.31
509	2664	62	0	325	1316	1414.5	1463	2277	2073	99.56
674	2130	34	0	197	1311	1436.3	1463	1899	1095	99.21
396	2246	38	0	106	1332	1407.0	1462	2102	1953	99.05
399	2317	45	1	47	1323	1420.0	1465	2224	2120	98.65
462	2349	47	0	394	1312	1417.5	1478	1908	1794	99.27
501	2246	22	0	253	1328	1442.9	1491	1971	1949	99.04
519	2062	51	0	297	1323	1414.5	1534	1714	1632	99.71
636	2402	35	0	100	1313	1409.7	1478	2267	2206	99.07
388	2454	78	1	78	1326	1406.6	1464	2297	1929	99.26
504	2312	29	0	284	1335	1409.3	1446	1999	1945	99.60
505	2702	45	0	48	1331	1415.2	1475	2609	2497	99.46
508	2380	30	1	210	1329	1436.5	1478	2139	2133	99.02

Supplementary Table S2 – PERMANOVA test results of the microbial community of Egyptian mongoose comparison between female and male and between non-adult and adult. N.S. = non-significant difference. p = p-value.

	UniFrac	Generalized Unifrac	Bray-Curtis	Jensen-Shannon
Female Vs. Male	N.S. (p=0.989)	N.S. (p=0.997)	N.S. (p=0.991)	N.S. (p=0.986)
Non-adult Vs. Adult	N.S. (p=0.581)	N.S. (p=0.499)	N.S. (p=0.439)	N.S. (p=0.350)

Supplementary Table S3 – Taxonomic biomarkers discovery of female and male Egyptian mongoose fecal bacterial microbiota using a Kruskal-Wallis H test. FDR = False-discovery rate.

Taxon name	Taxon rank	Taxonomy	p-value	p-value (FDR)	Female	Male
Kocuria	Genus	Bacteria: Actinobacteria: Actinobacteria_c: Micrococcales: Micrococcaceae	0.03057	0.03057	0.00000	0.09820
Kocuria rhizophila	Species	Bacteria: Actinobacteria: Actinobacteria_c: Micrococcales: Micrococcaceae: Kocuria	0.03057	0.03061	0.00000	0.09291
Hathewaya	Genus	Bacteria: Firmicutes: Clostridia: Clostridiales: Clostridiaceae	0.03057	0.03066	0.00000	0.71680
Hathewaya limosa	Species	Bacteria: Firmicutes: Clostridia: Clostridiales: Clostridiaceae: Hathewaya	0.03057	0.03070	0.00000	0.71680
LT594785_s	Species	Bacteria: Firmicutes: Clostridia: Clostridiales: Clostridiaceae: Clostridium	0.03057	0.03074	0.46272	0.00000
Clostridium haemolyticum group	Species	Bacteria: Firmicutes: Clostridia: Clostridiales: Clostridiaceae: Clostridium	0.03292	0.03316	0.02597	2.55440

Supplementary Table S4 – Taxonomic biomarkers discovery of non-adult and adult Egyptian mongoose fecal bacterial microbiota using a Kruskal-Wallis H test. FDR = False-discovery rate.

Taxon name	Taxon rank	Taxonomy	p-value	p-value (FDR)	Non-adult	Adult
Erysipelotrichales_uc	Family	Bacteria: Firmicutes: Erysipelotrichi: Erysipelotrichales	0.00371	0.00371	0.92817	0.00000
EU462708_g	Genus	Bacteria: Firmicutes: Clostridia: Clostridiales: Eubacteriaceae	0.00371	0.00371	0.04798	0.00000
Kurthia gibsonii	Species	Bacteria: Firmicutes: Bacilli: Bacillales: Planococcaceae: Kurthia	0.00371	0.00372	0.04533	0.00000
EU462708_s	Species	Bacteria: Firmicutes: Clostridia: Clostridiales: Eubacteriaceae: EU462708_g	0.00371	0.00372	0.04798	0.00000
LN849007_s	Species	Bacteria: Firmicutes: Clostridia: Clostridiales: Clostridiaceae: Clostridium	0.00371	0.00373	0.03350	0.00000
Clostridium septicum	Species	Bacteria: Firmicutes: Clostridia: Clostridiales: Clostridiaceae: Clostridium	0.00429	0.00432	0.37781	0.00660
Clostridium novyi	Species	Bacteria: Firmicutes: Clostridia: Clostridiales: Clostridiaceae: Clostridium	0.00876	0.00884	1.23108	0.02009
Eubacteriaceae	Family	Bacteria: Firmicutes: Clostridia: Clostridiales	0.01693	0.01710	1.51794	0.01424
Eubacteriaceae_uc	Genus	Bacteria: Firmicutes: Clostridia: Clostridiales: Eubacteriaceae	0.02261	0.02287	1.42642	0.00562
LFHY_s	Species	Bacteria: Proteobacteria: Gammaproteobacteria: Enterobacterales: Enterobacteriaceae: Escherichia	0.02288	0.02317	0.91041	0.48837
Clostridium_g6	Genus	Bacteria: Firmicutes: Erysipelotrichi: Erysipelotrichales: Erysipelotrichaceae	0.02539	0.02576	0.04610	0.00952
Clostridium ramosum	Species	Bacteria: Firmicutes: Erysipelotrichi: Erysipelotrichales: Erysipelotrichaceae: Clostridium_g6	0.02539	0.02580	0.04610	0.00952
Paraclostridium benzoelyticum group	Species	Bacteria: Firmicutes: Clostridia: Clostridiales: Peptostreptococcaceae: Paraclostridium	0.03115	0.03168	0.18578	0.03920
Glutamicibacter	Genus	Bacteria: Actinobacteria: Actinobacteria_c: Micrococcales: Micrococcaceae	0.03334	0.03396	0.09890	0.00565
Tissierellia	Class	Bacteria: Firmicutes	0.03334	0.03401	0.13103	0.01984
Tissierellales	Order	Bacteria: Firmicutes: Tissierellia	0.03334	0.03406	0.13103	0.01984
Slackia	Genus	Bacteria: Actinobacteria: Coriobacteriia: Coriobacteriales: Coriobacteriaceae	0.03764	0.03851	1.49037	0.85319
Slackia faecicanis	Species	Bacteria: Actinobacteria: Coriobacteriia: Coriobacteriales: Coriobacteriaceae: Slackia	0.03764	0.03856	1.49037	0.83122
PAC001163_s	Species	Bacteria: Firmicutes: Clostridia: Clostridiales: Lachnospiraceae: Blautia	0.04262	0.04373	0.94146	0.23776
Leclercia	Genus	Bacteria: Proteobacteria: Gammaproteobacteria: Enterobacterales: Enterobacteriaceae	0.04550	0.04675	0.02177	0.00000
Marmoricola	Genus	Bacteria: Actinobacteria: Actinobacteria_c: Propionibacteriales: Nocardioidaceae	0.04550	0.04682	0.01088	0.00000
EU462644_g	Genus	Bacteria: Firmicutes: Clostridia: Clostridiales: Eubacteriaceae	0.04550	0.04689	0.04354	0.00000
Corynebacterium efficiens	Species	Bacteria: Actinobacteria: Actinobacteria_c: Corynebacteriales: Corynebacteriaceae: Corynebacterium	0.04550	0.04696	0.01088	0.00000

Corynebacterium mastitidis	Species	Bacteria: Actinobacteria: Actinobacteria_c: Corynebacteriales: Corynebacteriaceae: Corynebacterium	0.04550	0.04703	0.01088	0.00000
Glutamicibacter bergerei group	Species	Bacteria: Actinobacteria: Actinobacteria_c: Micrococcales: Micrococcaceae: Glutamicibacter	0.04550	0.04710	0.06530	0.00000
Leclercia adecarboxylata group	Species	Bacteria: Proteobacteria: Gammaproteobacteria: Enterobacterales: Enterobacteriaceae: Leclercia	0.04550	0.04717	0.02177	0.00000
Leucobacter aerolatus	Species	Bacteria: Actinobacteria: Actinobacteria_c: Micrococcales: Microbacteriaceae: Leucobacter	0.04550	0.04723	0.01088	0.00000
Marmoricola aequoreus	Species	Bacteria: Actinobacteria: Actinobacteria_c: Propionibacteriales: Nocardioidaceae: Marmoricola	0.04550	0.04730	0.01088	0.00000
Paeniglutamicibacter sulfureus	Species	Bacteria: Actinobacteria: Actinobacteria_c: Micrococcales: Micrococcaceae: Paeniglutamicibacter	0.04550	0.04737	0.03265	0.00000
JF016390_s	Species	Bacteria: Actinobacteria: Thermoleophilia: Solirubrobacterales: Conexibacteraceae: Conexibacter	0.04550	0.04744	0.01088	0.00000
EU462644_g_uc	Species	Bacteria: Firmicutes: Clostridia: Clostridiales: Eubacteriaceae: EU462644_g	0.04550	0.04751	0.04354	0.00000
Clostridia_uc	Order	Bacteria: Firmicutes: Clostridia	0.04550	0.04758	0.08278	0.00000
Cellulomonadaceae	Family	Bacteria: Actinobacteria: Actinobacteria_c: Micrococcales	0.04550	0.04765	0.01183	0.00000
Rikenellaceae	Family	Bacteria: Bacteroidetes: Bacteroidia: Bacteroidales	0.04550	0.04772	0.01183	0.00000
Alistipes	Genus	Bacteria: Bacteroidetes: Bacteroidia: Bacteroidales: Rikenellaceae	0.04550	0.04780	0.01183	0.00000
Anaerostipes	Genus	Bacteria: Firmicutes: Clostridia: Clostridiales: Lachnospiraceae	0.04550	0.04787	0.02365	0.00000
Cellulomonas	Genus	Bacteria: Actinobacteria: Actinobacteria_c: Micrococcales: Cellulomonadaceae	0.04550	0.04794	0.01183	0.00000
Dorea	Genus	Bacteria: Firmicutes: Clostridia: Clostridiales: Lachnospiraceae	0.04550	0.04801	0.01183	0.00000
Frisingicoccus	Genus	Bacteria: Firmicutes: Clostridia: Clostridiales: Lachnospiraceae	0.04550	0.04808	0.02365	0.00000
Monoglobus	Genus	Bacteria: Firmicutes: Clostridia: Clostridiales: Ruminococcaceae	0.04550	0.04815	0.02365	0.00000
Tetragenococcus	Genus	Bacteria: Firmicutes: Bacilli: Lactobacillales: Enterococcaceae	0.04550	0.04822	0.01183	0.00000
PAC001200_g	Genus	Bacteria: Firmicutes: Clostridia: Clostridiales: Lachnospiraceae	0.04550	0.04830	0.01183	0.00000
Saccharimonas_f_uc	Genus	Bacteria: Saccharibacteria_TM7: Saccharimonas_c: Saccharimonas_o: Saccharimonas_f	0.04550	0.04837	0.03548	0.00000
Alistipes putredinis	Species	Bacteria: Bacteroidetes: Bacteroidia: Bacteroidales: Rikenellaceae: Alistipes	0.04550	0.04844	0.01183	0.00000
Anaerostipes caccae	Species	Bacteria: Firmicutes: Clostridia: Clostridiales: Lachnospiraceae: Anaerostipes	0.04550	0.04852	0.02365	0.00000
Cellulomonas composti	Species	Bacteria: Actinobacteria: Actinobacteria_c: Micrococcales: Cellulomonadaceae: Cellulomonas	0.04550	0.04859	0.01183	0.00000
Clostridium cadaveris	Species	Bacteria: Firmicutes: Clostridia: Clostridiales: Clostridiaceae: Clostridium	0.04550	0.04866	0.01183	0.00000
Corynebacterium callunae	Species	Bacteria: Actinobacteria: Actinobacteria_c: Corynebacteriales: Corynebacteriaceae: Corynebacterium	0.04550	0.04874	0.01183	0.00000
Corynebacterium variabile	Species	Bacteria: Actinobacteria: Actinobacteria_c: Corynebacteriales: Corynebacteriaceae: Corynebacterium	0.04550	0.04881	0.05913	0.00000
Dorea massiliensis	Species	Bacteria: Firmicutes: Clostridia: Clostridiales: Lachnospiraceae: Dorea	0.04550	0.04888	0.01183	0.00000

Glutamicibacter soli	Species	Bacteria: Actinobacteria: Actinobacteria_c: Micrococcales: Micrococcaceae: Glutamicibacter	0.04550	0.04896	0.01183	0.00000
Lactobacillus gasseri group	Species	Bacteria: Firmicutes: Bacilli: Lactobacillales: Lactobacillaceae: Lactobacillus	0.04550	0.04903	0.01183	0.00000
Lactobacillus pontis	Species	Bacteria: Firmicutes: Bacilli: Lactobacillales: Lactobacillaceae: Lactobacillus	0.04550	0.04911	0.01183	0.00000
Streptococcus parauberis	Species	Bacteria: Firmicutes: Bacilli: Lactobacillales: Streptococcaceae: Streptococcus	0.04550	0.04918	0.01183	0.00000
Tetragenococcus halophilus group	Species	Bacteria: Firmicutes: Bacilli: Lactobacillales: Enterococcaceae: Tetragenococcus	0.04550	0.04926	0.01183	0.00000
Tissierella praeacuta	Species	Bacteria: Firmicutes: Tissierellia: Tissierellales: Tissierellaceae: Tissierella	0.04550	0.04933	0.01183	0.00000
CP017156_s	Species	Bacteria: Firmicutes: Bacilli: Bacillales: Staphylococcaceae: Macrococcus	0.04550	0.04941	0.01183	0.00000
EU458641_s	Species	Bacteria: Firmicutes: Clostridia: Clostridiales: Ruminococcaceae: Monoglobus	0.04550	0.04948	0.02365	0.00000
EU462940_s	Species	Bacteria: Firmicutes: Clostridia: Clostridiales: Lachnospiraceae: PAC001200_g	0.04550	0.04956	0.01183	0.00000
EU776034_s	Species	Bacteria: Firmicutes: Clostridia: Clostridiales: Lachnospiraceae: Blautia	0.04550	0.04964	0.02365	0.00000
FJ538159_s	Species	Bacteria: Actinobacteria: Actinobacteria_c: Streptosporangiales: AF498716_f: PAC000166_g	0.04550	0.04971	0.01183	0.00000
HQ743744_s	Species	Bacteria: Firmicutes: Clostridia: Clostridiales: Lachnospiraceae: Blautia	0.04550	0.04979	0.39026	0.00000
Faecalimonas_uc	Species	Bacteria: Firmicutes: Clostridia: Clostridiales: Lachnospiraceae: Faecalimonas	0.04550	0.04987	0.02365	0.00000
Frisingicoccus_uc	Species	Bacteria: Firmicutes: Clostridia: Clostridiales: Lachnospiraceae: Frisingicoccus	0.04550	0.04994	0.02365	0.00000
Peptoniphilaceae	Family	Bacteria: Firmicutes: Tissierellia: Tissierellales	0.04550	0.05002	0.05418	0.00000
Nosocomiicoccus	Genus	Bacteria: Firmicutes: Bacilli: Bacillales: Staphylococcaceae	0.04550	0.05010	0.01084	0.00000
Peptoniphilus	Genus	Bacteria: Firmicutes: Tissierellia: Tissierellales: Peptoniphilaceae	0.04550	0.05018	0.01084	0.00000
AB298774_g	Genus	Bacteria: Firmicutes: Tissierellia: Tissierellales: Peptoniphilaceae	0.04550	0.05026	0.03251	0.00000
AM500828_g	Genus	Bacteria: Firmicutes: Tissierellia: Tissierellales: Peptoniphilaceae	0.04550	0.05033	0.01084	0.00000
Bacillaceae_uc	Genus	Bacteria: Firmicutes: Bacilli: Bacillales: Bacillaceae	0.04550	0.05041	0.02167	0.00000
Tissierellaceae_uc	Genus	Bacteria: Firmicutes: Tissierellia: Tissierellales: Tissierellaceae	0.04550	0.05049	0.06502	0.00000
Clostridium massiliodielmoense	Species	Bacteria: Firmicutes: Clostridia: Clostridiales: Clostridiaceae: Clostridium	0.04550	0.05057	0.17339	0.00000
Corynebacterium ammoniagenes	Species	Bacteria: Actinobacteria: Actinobacteria_c: Corynebacteriales: Corynebacteriaceae: Corynebacterium	0.04550	0.05065	0.01084	0.00000
Nosocomiicoccus ampullae	Species	Bacteria: Firmicutes: Bacilli: Bacillales: Staphylococcaceae: Nosocomiicoccus	0.04550	0.05073	0.01084	0.00000
Peptoniphilus stercorisuis	Species	Bacteria: Firmicutes: Tissierellia: Tissierellales: Peptoniphilaceae: Peptoniphilus	0.04550	0.05081	0.01084	0.00000
Staphylococcus sciuri	Species	Bacteria: Firmicutes: Bacilli: Bacillales: Staphylococcaceae: Staphylococcus	0.04550	0.05089	0.01084	0.00000
Vagococcus carniphilus	Species	Bacteria: Firmicutes: Bacilli: Lactobacillales: Enterococcaceae: Vagococcus	0.04550	0.05097	0.04335	0.00000

Vagococcus humatus	Species	Bacteria: Firmicutes: Bacilli: Lactobacillales: Enterococcaceae: Vagococcus	0.04550	0.05105	0.01084	0.00000
AB298774_s	Species	Bacteria: Firmicutes: Tissierellia: Tissierellales: Peptoniphilaceae: AB298774_g	0.04550	0.05113	0.03251	0.00000
AM500828_s	Species	Bacteria: Firmicutes: Tissierellia: Tissierellales: Peptoniphilaceae: AM500828_g	0.04550	0.05121	0.01084	0.00000
Ruminococcus_g5_uc	Species	Bacteria: Firmicutes: Clostridia: Clostridiales: Lachnospiraceae: Ruminococcus_g5	0.04550	0.05130	0.02167	0.00000
Peptostreptococcaceae_uc	Genus	Bacteria: Firmicutes: Clostridia: Clostridiales: Peptostreptococcaceae	0.04550	0.05138	0.02501	0.00000
Clostridium fallax	Species	Bacteria: Firmicutes: Clostridia: Clostridiales: Clostridiaceae: Clostridium	0.04550	0.05146	0.01251	0.00000
Escherichia_uc	Species	Bacteria: Proteobacteria: Gammaproteobacteria: Enterobacterales: Enterobacteriaceae: Escherichia	0.04816	0.05455	0.02172	0.00328
Escherichia marmotae	Species	Bacteria: Proteobacteria: Gammaproteobacteria: Enterobacterales: Enterobacteriaceae: Escherichia	0.04816	0.05464	0.02339	0.01694
Tissierellaceae	Family	Bacteria: Firmicutes: Tissierellia: Tissierellales	0.04816	0.05473	0.07685	0.01984

Supplementary Table S5 – Functional biomarkers discovery of female and male Egyptian mongoose fecal bacterial microbiota applying a LEfSe analysis. LDA = linear discriminant analysis. FDR = False-discovery rate.

Ortholog	Definition	LDA effect size	p-value	p-value (FDR)	Female	Male
K00102	D-lactate dehydrogenase (cytochrome)	182767	0.00650	0.00651	0.01462	0.02787
K03650	tRNA modification GTPase	180642	0.00650	0.00651	0.06544	0.07805
K04717	sphingosine-1-phosphate phosphotase 2	188296	0.00650	0.00651	0.01992	0.03500
K00752	hyaluronan synthase	189263	0.00815	0.00817	0.02430	0.03972
K13292	phosphatidylglycerol---prolipoprotein diacylglyceryl transferase	195539	0.01261	0.01268	0.06917	0.05132
K00678	phosphatidylcholine-retinol O-acyltransferase	205743	0.01911	0.01929	0.02608	0.04871
K02777	PTS system, sugar-specific IIA component	183824	0.01911	0.01934	0.04337	0.02979
K06817	podocalyxin-like	185618	0.01911	0.01930	0.01625	0.03042
K16345	xanthine permease XanP	179350	0.02334	0.02370	0.01369	0.02592
K19302	undecaprenyl-diphosphatase	179809	0.02334	0.02371	0.07894	0.06657
K02018	molybdate transport system permease protein	189352	0.02837	0.02885	0.03921	0.05467
K03475	PTS system, ascorbate-specific IIC component	184732	0.02837	0.02891	0.03981	0.02594
K02020	molybdate transport system substrate-binding protein	201408	0.03429	0.03519	0.04576	0.06622
K03567	glycine cleavage system transcriptional repressor	179535	0.03429	0.03515	0.01835	0.03063
K04456	RAC serine/threonine-protein kinase	178343	0.03429	0.03518	0.01415	0.02610
K01185	lysozyme	218217	0.04125	0.04242	0.05956	0.02933
K03111	single-strand DNA-binding protein	190148	0.04937	0.05103	0.12586	0.11012
K03300	citrate-Mg2+:H+ or citrate-Ca2+:H+ symporter, CitMHS family	181420	0.04937	0.05103	0.02086	0.03370
K03303	lactate permease	196174	0.04937	0.05100	0.03879	0.05690
K03537	ribonuclease P/MRP protein subunit POP5	177067	0.04937	0.05108	0.01585	0.02744
K04072	acetaldehyde dehydrogenase / alcohol dehydrogenase	176491	0.04937	0.05107	0.05179	0.04035
K06129	lysophospholipase III	180406	0.04937	0.05115	0.01616	0.02870
K06984	beta-ribofuranosylaminobenzene 5'-phosphate synthase	204943	0.04937	0.05116	0.03236	0.05457
Module	Definition	LDA effect size	p-value	p-value (FDR)	Female	Male

M00173	Reductive citrate cycle (Arnon-Buchanan cycle)	296692	0.02837	0.02924	0.89145	1.07658
M00011	Citrate cycle, second carbon oxidation, 2-oxoglutarate => oxaloacetate	302014	0.03429	0.03549	0.58289	0.79217
Pathway	Definition	LDA effect size	p-value	p-value (FDR)	Female	Male
ko00380	Tryptophan metabolism	255955	0.03429	0.03678	0.17318	0.24551
ko00052	Galactose metabolism	283596	0.04937	0.05373	0.81785	0.68096
ko00280	Valine, leucine and isoleucine degradation	262758	0.04937	0.05427	0.24041	0.32504

Supplementary Table S6 – Functional biomarkers discovery of non-adult and adult Egyptian mongoose fecal bacterial microbiota applying a LEfSe analysis. LDA = linear discriminant analysis. FDR = False-discovery rate.

Ortholog	Definition	LDA effect size	p-value	p-value (FDR)	Non-adult	Adult
K07487	transposase	226521	0.00614	0.00615	0.05943	0.02280
K02172	bla regulator protein blaR1	208451	0.01402	0.01406	0.05653	0.03243
K20487	two-component system, OmpR family, lantibiotic biosynthesis sensor histidine kinase NisK/SpaK	200890	0.01402	0.01406	0.05709	0.03688
K00845	glucokinase	210077	0.01402	0.01407	0.11460	0.13962
K08083	two-component system, LytTR family, response regulator AlgR	204416	0.01816	0.01825	0.06464	0.04270
K19294	alginate O-acetyltransferase complex protein AlgI	198546	0.01816	0.01826	0.05643	0.03729
K00548	5-methyltetrahydrofolate--homocysteine methyltransferase	232270	0.01816	0.01826	0.08541	0.04356
K02406	flagellin	191690	0.02334	0.02349	0.03178	0.01546
K07012	CRISPR-associated endonuclease/helicase Cas3	190424	0.02334	0.02350	0.03274	0.01690
K01785	aldose 1-epimerase	210543	0.02334	0.02351	0.05428	0.07957
K15580	oligopeptide transport system substrate-binding protein	216873	0.02334	0.02351	0.04270	0.07200
K03522	electron transfer flavoprotein alpha subunit	209704	0.02976	0.03007	0.09436	0.06955
K07258	serine-type D-Ala-D-Ala carboxypeptidase (penicillin-binding protein 5/6)	208349	0.02976	0.03007	0.15519	0.13115
K04069	pyruvate formate lyase activating enzyme	230244	0.02976	0.03008	0.14147	0.10154
K03616	electron transport complex protein RnfB	196117	0.03764	0.03820	0.06807	0.04998
K07814	putative two-component system response regulator	223738	0.03764	0.03822	0.05556	0.02122
K05910	NADH peroxidase	193555	0.03764	0.03831	0.00665	0.02369
K03406	methyl-accepting chemotaxis protein	271229	0.04722	0.04856	0.19960	0.09668
K18346	vancomycin resistance protein VanW	196209	0.04722	0.04858	0.05372	0.03559
Module	Definition	LDA effect size	p-value	p-value (FDR)	non-adults	adults
M00725	Cationic antimicrobial peptide (CAMP) resistance, dltABCD operon	258995	0.03764	0.03813	0.06132	0.13892
Pathway	Definition	LDA effect size	p-value	p-value (FDR)	non-adults	adults
ko02020	Two-component system	299991	0.04722	0.04809	272432	252526

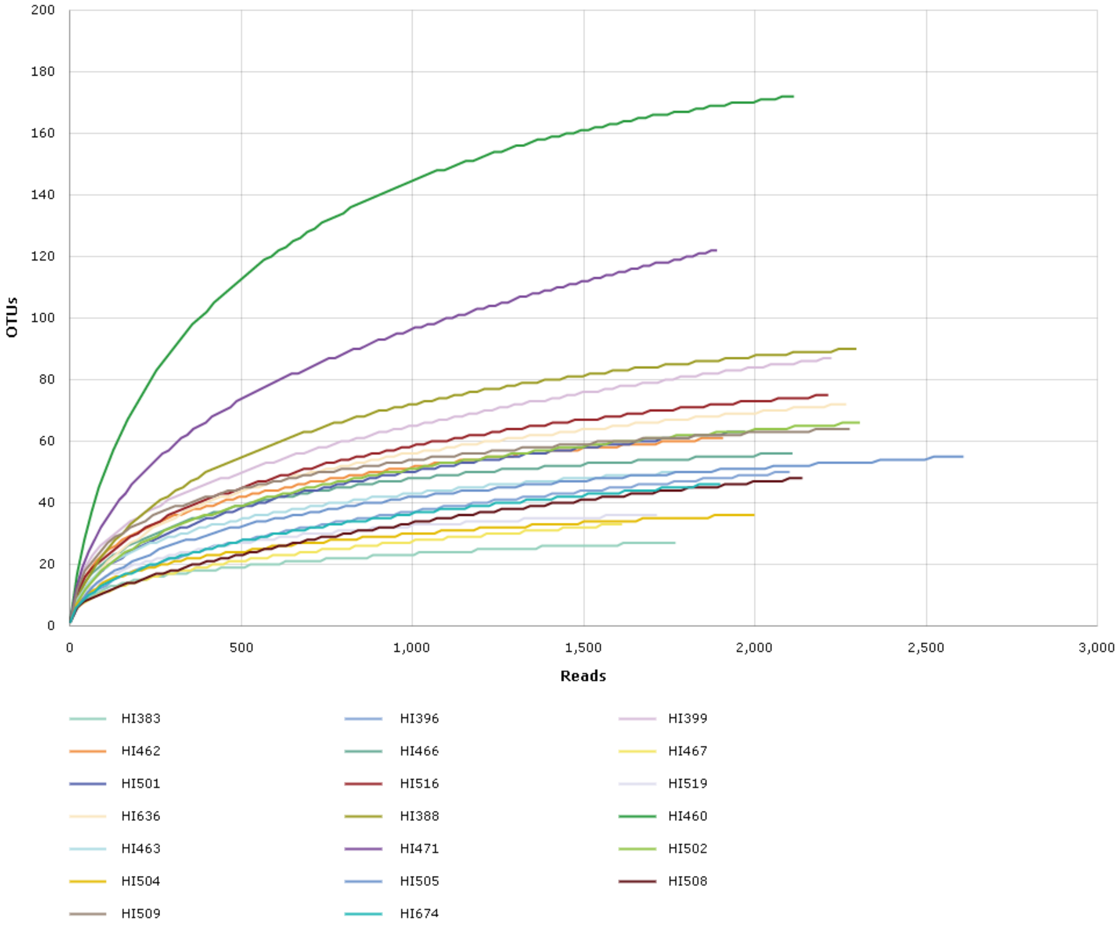

Supplementary Figure S1. Rarefaction curves for the twenty different fecal samples of Egyptian mongoose measured individuals using the observed species metric.

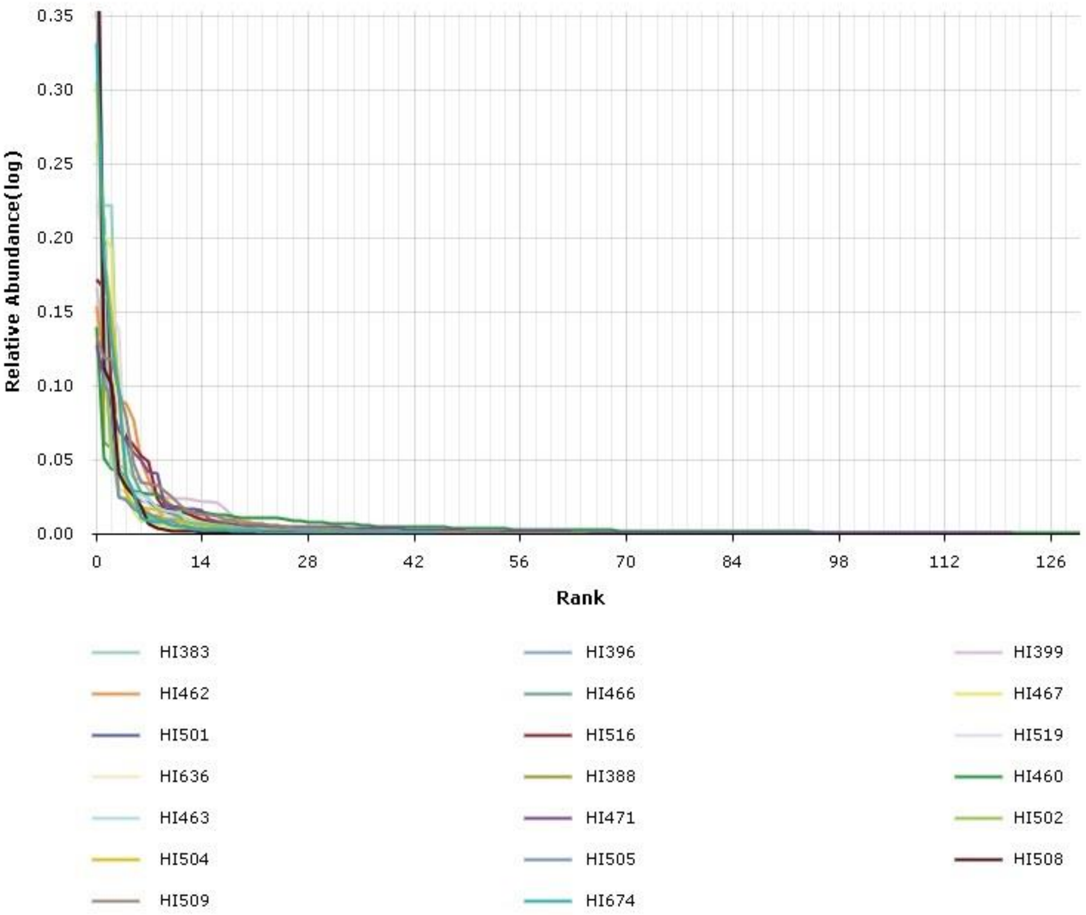

Supplementary Figure S2. Rank abundance curves for the twenty different fecal samples of Egyptian mongoose individuals using the observed species metric.

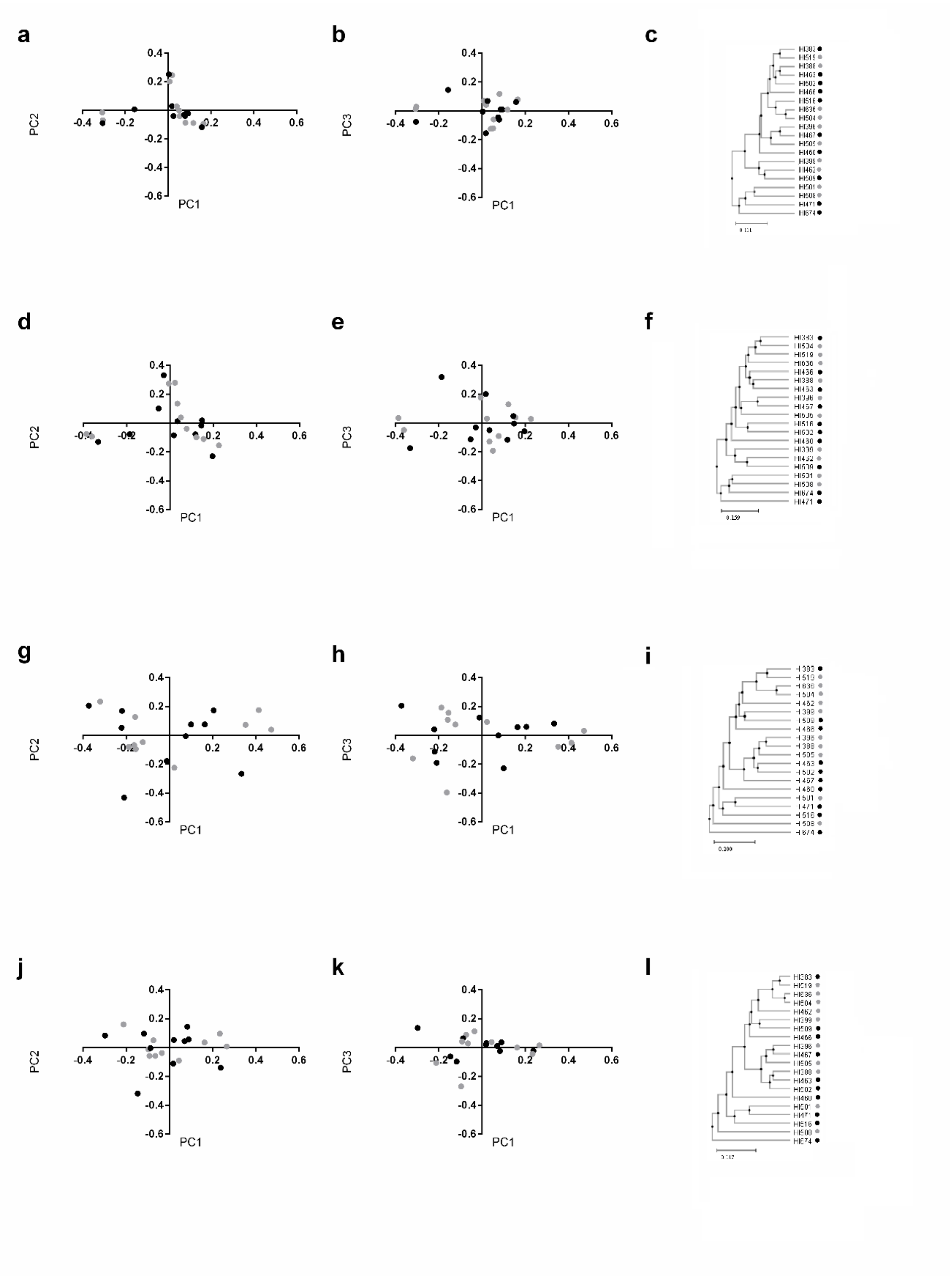

Supplementary Figure S3. Beta-diversity analysis to compare the fecal bacterial microbiota, at a genus level, of male and female Egyptian mongoose. Principal Coordinate Analysis of UniFrac distances (**a,b**), Generalized UniFrac distances (**d,e**), Bray-Curtis dissimilarities (**g,h**), and Jensen-Shannon distances (**j,k**). Dendrogram representing the relationship between the fecal bacterial microbiota of the twenty specimens that were clustered using UPGMA applied to the matrixes of UniFrac distances (**c**), Generalized UniFrac distances (**f**), Bray-Curtis dissimilarities (**i**), and Jensen-Shannon distances (**l**). Black dotes represent males and gray dotes represent females.

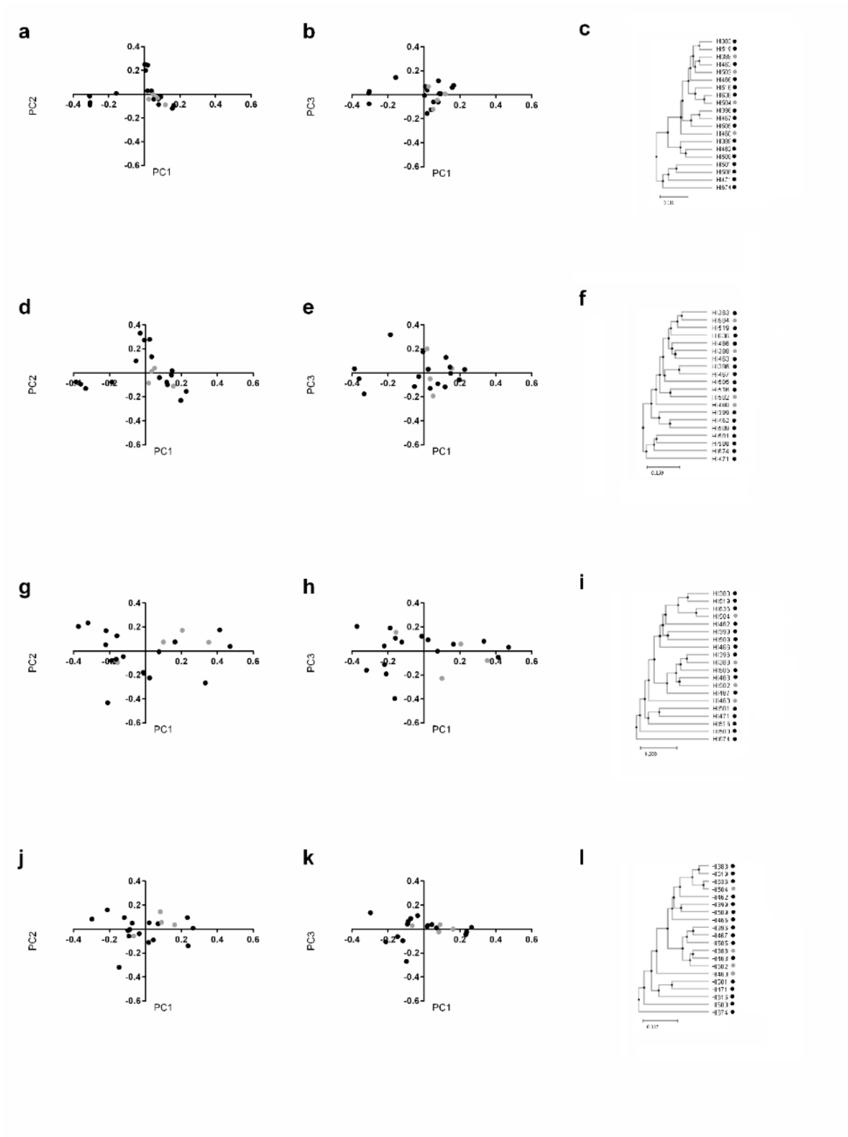

Supplementary Figure S4. Beta-diversity analysis to compare the fecal bacterial microbiota, at a genus level, of adult and non-adult Egyptian mongoose. Principal Coordinate Analysis of UniFrac distances (**a,b**), Generalized UniFrac distances (**d,e**), Bray-Curtis dissimilarities (**g,h**), and Jensen-Shannon distances (**j,k**). Dendrogram representing the relationship between the fecal bacterial microbiota of the twenty specimens that were clustered using UPGMA applied to the matrixes of UniFrac distances (**c**), Generalized UniFrac distances (**f**), Bray-Curtis dissimilarities (**i**), and Jensen-Shannon distances (**l**). Black dotes represent adults and gray dotes represent non-adults.
